# Tumor-Educated Platelets as a Promising Biomarker for Blood-Based Detection of Renal Cell Carcinoma

**DOI:** 10.3389/fonc.2022.844520

**Published:** 2022-03-07

**Authors:** Ruotao Xiao, Cheng Liu, Bo Zhang, Lulin Ma

**Affiliations:** ^1^ Department of Urology, Peking University Third Hospital, Beijing, China; ^2^ Department of Pathology, School of Basic Medical Sciences, Peking University Health Science Center, Beijing, China

**Keywords:** renal cell carcinoma, tumor-educated platelets, biomarkers, diagnosis, liquid biopsy

## Abstract

**Purpose:**

Tumor-educated platelets (TEPs) are a promising liquid biopsy in many cancers. However, their role in renal cell carcinoma (RCC) is unknown. Thus, this study explored the diagnostic value of TEPs in RCC patients.

**Methods:**

Platelets were prospectively collected from 24 RCC patients and 25 controls. RNA-seq was performed to identify the differentially expressed genes (DEGs) between RCC patients and controls. Besides, RNA-seq data of pan-cancer TEPs were downloaded and randomly divided into training and validation sets. A pan-cancer TEP model was developed in the training set using the support vector machine (SVM) and validated in the validation set and our RCC dataset. Finally, an RCC-based TEP model was developed and optimized through the SVM algorithms and recursive feature elimination (RFE) method.

**Result:**

Two hundred three DEGs, 64 (31.5%) upregulated and 139 (68.5%) downregulated, were detected in the platelets of RCC patients compared with controls. The pan-cancer TEP model had a high accuracy in detecting cancer in the internal validation (training set, accuracy 98.8%, AUC: 0.999; validation set, accuracy 95.4%, AUC: 0.972; different tumor subtypes, accuracy 86.6%–96.1%, AUC: 0.952–1.000). However, the pan-cancer TEP model in the external validation had a scarce diagnostic value in RCC patients (accuracy 48.7%, AUC: 0.615). Therefore, to develop the RCC-based TEP model, the gene biomarkers mostly contributing to the model were selected using the RFE method. The RCC-based TEP model containing 68 gene biomarkers reached a diagnostic accuracy of 100% (AUC: 1.000) in the training set, 88.9% (AUC: 0.963) in the validation set, and 95.9% (AUC: 0.988) in the overall cohort.

**Conclusion:**

TEPs could function as a minimally invasive blood biomarker in the detection of RCC.

## Introduction

Renal cell carcinoma (RCC) is the 10th most frequently diagnosed cancer worldwide accounting for more than 90% of kidney tumors (1, 2). It is derived from the epithelial cells of the renal tubules. Localized RCC has a relatively favorable oncologic outcome after curative surgery, with a 5-year cancer-specific survival rate of 71%~88% (3). However, approximately 20%~30% of patients already have metastasis at the time of diagnosis, with a median survival less than 2 years after cytoreductive surgery and adjuvant therapy (4, 5). Therefore, early detection of RCC is associated with a higher possibility of resecting the tumor, obtaining a better survival outcome. Blood-based tumor biomarkers have been developed in many cancers for screening and monitoring the tumor, such as alpha-fetoprotein in hepatocellular carcinoma, carcinoembryonic antigen in colorectal cancer, and prostate-specific antigen in prostate cancer (6–8). However, up to now, the diagnosis of RCC is still performed by the examination of images from the ultrasound scan, computed tomography, and magnetic resonance. Thus, blood-based biomarkers for the early diagnosis of RCC, as well as its screening and longitudinal monitoring, are still lacking.

Diagnostic tools for non-invasive tumors have rapidly developed in recent years, generating a new domain in cancer research called “liquid biopsy.” Liquid biopsy allows to obtain information on the tumor through the analysis of human blood, urine, cerebrospinal fluid, and other body fluids, so as to obtain an early diagnosis and monitoring of the tumors (9). The markers analyzed in these samples, such as circulating tumor cells, circulating tumor DNA, circulating tumor RNA, and exosomes, have been widely studied in many cancers (10–13). However, the practical application of such markers for the non-invasive detection of RCC has been hampered by their low accuracy and high cost (14, 15). Therefore, the investigation of more cost-effective approaches is of paramount importance to enable urologists to diagnose RCC through a blood biopsy.

Accumulating evidence suggested the existence of a wide variety of cross-talk between platelets and tumor cells, promoting tumor progression and metastasis (16). Besides, tumor cells can modify the RNA profile of the platelets, indicating that the platelet transcriptome can be potentially used to diagnose cancer (16). Best et al. (17, 18) performed a series of studies on the platelet transcriptome, bringing a novel insight into the role of liquid biopsy. They indeed found that the platelets called tumor-educated platelets (TEPs), which are platelets modified by the tumor, can be used as a high-accuracy biomarker to distinguish patients with pan-cancer and non-small cell lung cancer (NSCLC) from healthy individuals (17, 18). The following studies further confirmed that TEPs can be used as a liquid biopsy in the diagnosis of sarcoma cancer, primary thyroid cancer (PTC), ovarian cancer, and glioblastoma (GBM) (19–22). However, there is still a lack of evidence to illustrate whether platelet RNA could also change in patients with RCC. Besides, the diagnostic value of TEPs in patients with RCC is still unknown. Therefore, the aim of this study was to characterize the transcriptome of TEPs from patients with RCC and controls to investigate whether they could be used as a blood-based biomarker in the detection of RCC.

## Material and Methods

### Sample Collection and Platelet Isolation

Patients diagnosed with RCC were prospectively recruited from the Peking University Third Hospital between October 2020 and May 2021 after the approval of the study by the Peking University Third Hospital Medical Science Research Ethics Committee (IRB:00006761-M2021003). In addition, patients with benign renal tumor (BN) and healthy donors (HD) were also recruited and used as the control group. The donors subjected to antiplatelet therapy or diagnosed with blood disease, acute infection, autoimmune disease, and severe renal and liver dysfunction were excluded from this study. Finally, 49 platelet samples from 24 patients with RCC, 12 patients with BN, and 13 HD were included in this study.

Platelets were obtained from the peripheral whole blood collected using 10 ml purple-cap vacutainers containing EDTA. Platelets were isolated within 24 h after blood collection to minimize their activation and RNA degeneration by two-step centrifugations at room temperature according to the protocol reported by Best et al. (23). The platelet pellet was harvested after centrifugation and resuspended in 1 ml TRIzol reagent. Finally, the platelet pellet was stored at −80°C for further analysis. Platelets were randomly collected and purity was assessed by morphological analysis, using the criteria of less than 1~5 leukocytes per one million platelets (23).

### RNA Preparation and Sequencing

RNA was extracted from the platelets and assessed using the RNA Nano 6000 Assay Kit of the Bioanalyzer 2100 system. A total amount of RNA ≥100 ng and integrity number ≥7 were used as input material for the RNA sample preparation. Briefly, mRNA was purified from the total RNA using the poly-T oligo-attached magnetic beads. Fragmentation was carried out using divalent cations under high temperature in First-Strand Synthesis Reaction Buffer (5×). The first-strand cDNA was synthesized using random hexamer primer and M-MuLV Reverse Transcriptase (RNase H−). The second-strand cDNA synthesis was subsequently performed using DNA Polymerase I and RNase H. The remaining overhangs were converted into blunt ends *via* exonuclease/polymerase activity. After adenylation of the 3′ ends of the DNA fragments, the adaptor with hairpin loop structure was ligated to prepare the adaptor for the hybridization. The library fragments were purified using the AMPure XP system (Beckman Coulter, Beverly, USA) to select cDNA fragments of a preferential length of 370~420 bp. The PCR was performed using Phusion High-Fidelity DNA polymerase, Universal PCR primers, and Index (X) Primer. The PCR products were purified by the AMPure XP system and the library quality was assessed on the Agilent Bioanalyzer 2100 system. The clustering of the index-coded samples was performed on a cBot Cluster Generation System using TruSeq PE Cluster Kit v3-cBot-HS (Illumina) according to the manufacturer’s instructions. After cluster generation, the library preparation was sequenced on an Illumina NovaSeq 6000 platform, and 150 bp paired-end reads were generated.

### Transcriptome Analysis

Raw reads in the fastq format were firstly processed through in-house perl scripts. In this step, clean reads were obtained by removing from raw data the reads containing the adaptor, those containing ploy-N, and reads of low quality. Thus, downstream analysis was performed on the clean data with high quality. Reference genome and gene model annotation files were downloaded directly from the genome website. The index of the reference genome was built using Hisat2 v2.0.5, and paired-end clean reads were aligned to the reference genome using Hisat2 v2.0.5. StringTie (1.3.3b) was used to assemble and annotate new transcripts for those genes that were not successfully mapped (24). The new transcripts were called “novel. number.”

The number of reads mapped to each gene was counted using Feature Counts v1.5.0-p3, and then the fragments per kilo base of transcript per million mapped fragments (FPKM) of each gene were calculated based on the length of the gene and read count mapped to this gene (25, 26). The differential expression of the genes in the two groups was evaluated using the edgeR package in R (3.22.5) (27). The *P*-values were adjusted using the Benjamini and Hochberg method (27). A false discovery rate (FDR) <0.05 and an absolute log2 fold change >1 were set as the threshold for a significant differential expression.

Gene Ontology (GO) enrichment analysis and Kyoto Encyclopedia of Genes and Genomes (KEGG) pathway analysis of the differentially expressed genes (DEGs) were performed by the cluster Profiler R package, in which the gene length bias was corrected. GO terms and KEGG pathways with FDR <0.05 were considered significantly enriched by the differentially expressed genes.

### Model Development and Validation Using the Support Vector Machine

The RNA-sequence data of pan-cancer and HD samples were downloaded from the Gene Expression Omnibus with the accession number GSE68086 (https://www.ncbi.nlm.nih.gov/geo/query/acc.cgi?acc=GSE68086). A total of 228 pan-cancer samples and 55 HD samples were available in the GSE68086 database, which were randomly divided into training and internal validation sets at a 6:4 ratio using the “caret” package in R. In addition, 1,072 DEGs reported by Best et al. (17) were selected as classification features to perform an internal validation. A support vector machine (SVM) algorithm (radial basis function, RBF) was developed for binary sample classification using the “e1072” package in R and optimized by the training set using a 10-fold internal cross-validation to determine the best gamma and cost parameters. After optimization, the SVM parameters were fixed and validated in both the training set and internal validation set, as well as in different tumor subtypes. The external validation in our dataset was performed by the overlap of the DEGs of GSE68086 and RCC, including the 1,051 DEGs that were normalized by FPKM and selected as classification features. The SVM algorithm was developed, optimized, and internally validated as described above. Then, the SVM algorithm was externally validated in the RCC dataset to determine whether the pan-cancer TEP model could be applied specifically to RCC patients.

Besides, our aim was to develop an effective TEP-based classifier for patients with RCC. For this purpose, our patients and controls were randomly divided into training and validation sets at a 6:4 ratio and the DEGs were identified as mentioned above. The recursive feature elimination (RFE) method was used to narrow down the features in the training group to further optimize the DEGs to the model (28). After optimization, the DEGs were selected as classification features. The development, optimization, and validation of the SVM model were achieved as described above.

**Figure 1:**
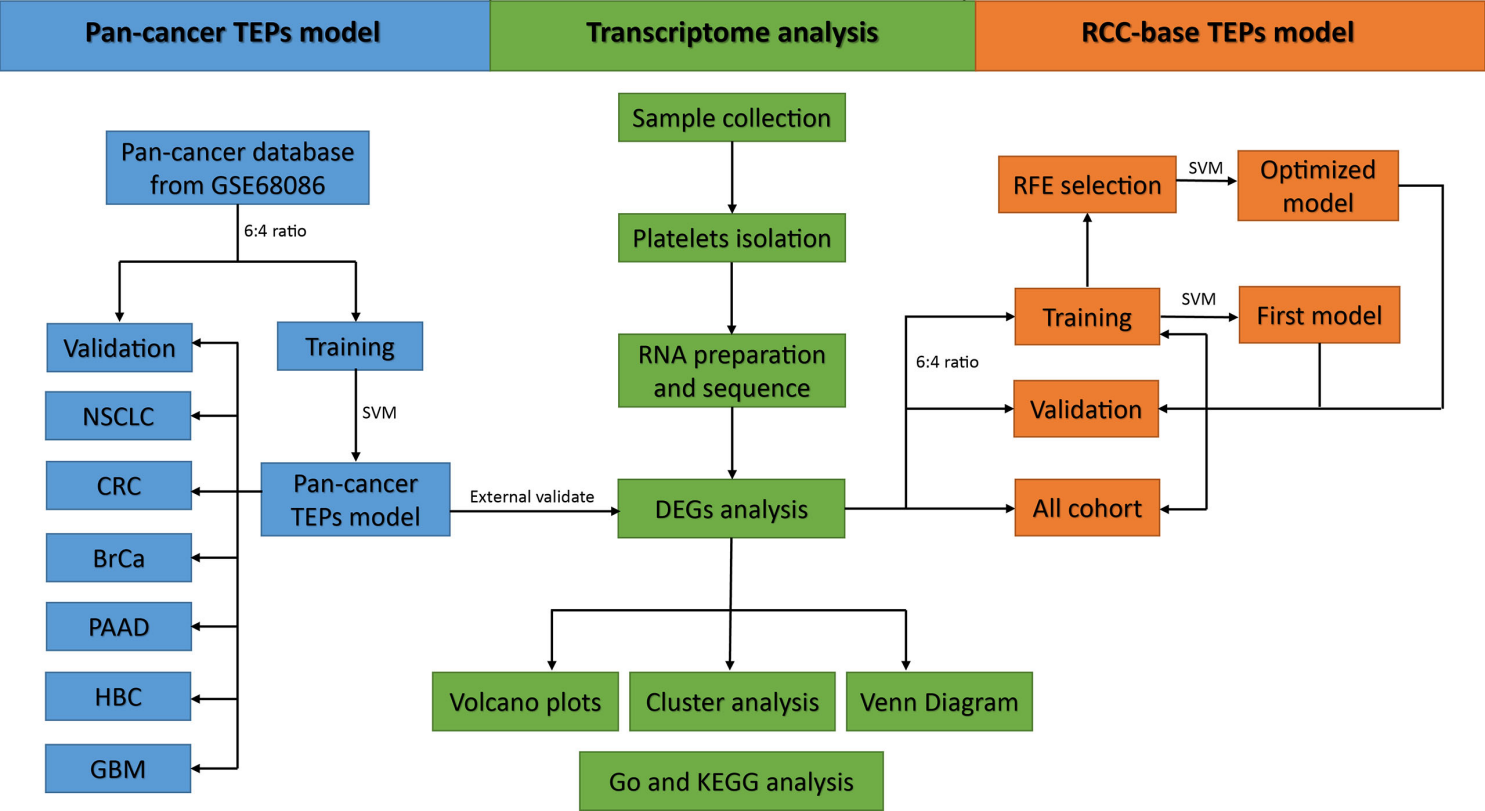
Flowchart of the main analytic steps in our study.

The predicted sample classes and actual sample classes were compared using the confounding matrix to measure the performance of the model by determining the sensitivity, specificity, and accuracy. The performance of the model was also evaluated by the receiver operating characteristic (ROC) curve and the area under the curve (AUC) using the “pROC” package in R. Overall, the flowchart of this study is summarized in **Figure 1**.

## Results

### Patients’ Characteristics

A total of 49 samples consisting of 24 RCC and 25 non-RCC control (13 HD, 12 BN) were used in this study. The baseline data including age, gender, BMI, complications, and laboratory indices between the RCC and control groups were similar, as shown in **Table 1**. A total of 18 cases, 2 cases, and 4 cases in the RCC group were clear cell renal cell carcinoma (ccRCC), papillary renal cell carcinoma (pRCC), and chromophobe renal cell carcinoma (chRCC), respectively (**Table 2**). Besides, 5 cases and 4 cases were combined with the involvement of lymph nodes and distant metastasis, respectively, at the time of diagnosis. Among the BN in the control group, 11 cases and 1 case were angiomyolipoma and oncocytoma, respectively. Platelets’ purity was assessed by morphological analysis, with satisfactory purity results (**Supplementary Figure 1**).

**Table 1:** The baseline data between the RCC and control groups.

Variables	Total (*N* = 49)	RCC (*N* = 24)	Control (*N* = 25)	*P*
Age, years	52.84 ± 12.05	53.82 ± 11.92	51.88 ± 12.34	0.567
Gender				
Men	25 (51)	14 (58.3)	11 (44)	0.396
Women	24 (49)	10 (41.7)	14 (56)	
BMI, kg/m^2^	24.69 ± 3.32	24.72 ± 2.91	24.66 ± 3.73	0.943
Complications				
Hypertension	17 (34.7)	10 (41.7)	7 (28)	0.377
Diabetes mellitus	8 (16.3)	3 (12.5)	5 (20)	0.702
Cardiovascular disease	5 (10.2)	3 (12.5)	2 (8)	0.667
Laboratory indices				
WBC, ×10^9^/L	6.58 ± 1.97	6.46 ± 2.28	6.70 ± 1.65	0.681
HGB, g/L	138.76 ± 19.17	140.67 ± 24.25	136.92 ± 12.82	0.506
PLT, ×10^9^/L	244.16 ± 77.16	248.08 ± 93.03	240.40 ± 59.85	0.731
MPV, fl	10.53 ± 1.2	10.33 ± 1.36	10.72 ± 1.01	0.259
PDW, fl	12.53 ± 2.75	12.09 ± 3.15	12.95 ± 2.29	0.281

BMI, body mass index; WBC, white blood cell; HGB, hemoglobin; PLT, platelet count; MPV, mean platelet volume; PDW, platelet distribution width.

**Table 2:** The pathological data of patients with RCC.

Variables	RCC patients (*N* = 24)
Tumor size, cm	6.61 ± 2.71
Histologic subtypes	
ccRCC	18 (75)
pRCC	2 (8.3)
chRCC	4 (16.7)
WHO/ISUP nuclear grade	
I–II	11 (45.8)
III–IV	9 (37.5)
Unknown	4 (16.7)
T stage	
T1–T2	6 (25)
T3–T4	18 (75)
N stage	
N0	19 (79.2)
N1	5 (20.8)
M stage	
M0	20 (83.3)
M1	4 (16.7)

ccRCC, clear cell renal cell carcinoma; pRCC, papillary renal cell carcinoma; chRCC, chromophobe renal cell carcinoma.

### DEGs Between the RCC and non-RCC Groups

**Figure 2:**
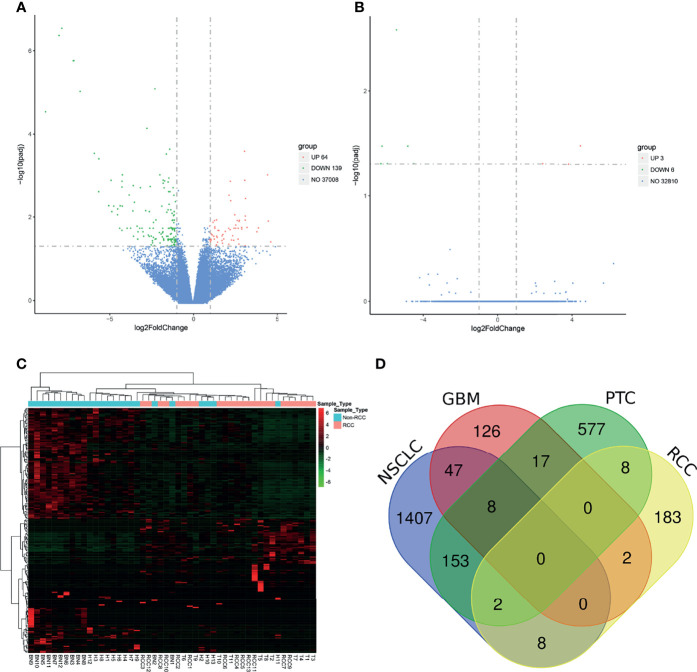
Analysis of differentially expressed genes (DEGs) in the platelet between the renal cell carcinoma (RCC) and control groups. **(A)** Volcano plot of DEGs between the RCC group and the control group. **(B)** Volcano plot of DEGs between groups A and B in the control group. **(C)** Unsupervised cluster analysis of DEGs in those platelet samples. **(D)** Venn diagram of DEGs in four different tumor types.

After the RNA-seq of the platelets of each sample, a mean read count of 40~50 million reads per sample was obtained. The general RNA expression patterns among individuals and among groups were similar (**Supplementary Figure 2**). Besides, the RNA profile of each sample in our dataset had a moderate to high correlation with the samples in the pan-cancer dataset (**Supplementary Table 1**). A total of 37,008 RNAs were detected in our platelet dataset. An FDR <0.05 and an absolute log2 fold change >1 were used as the statistical cutoffs to determine the DEGs between the RCC and non-RCC groups. Eventually, 203 DEGs were identified between the RCC and non-RCC groups, with 64 (31.5%) upregulated genes and 139 (68.5%) downregulated genes (**Figure 2A** and **Supplementary Table 2**). Furthermore, the non-RCC controls were randomly divided into A and B groups, but only 9 DEGs were identified between the A and B groups using the same criteria, suggesting that the DEGs between the RCC and non-RCC groups are mainly caused by cancer (**Figure 2B**). In subgroup analysis, the DEGs could also be identified in local RCC (59 DEGs) and metastatic RCC (39 DEGs) compared with controls (**Supplementary Figure 3**). Unsupervised hierarchical clustering analysis based on 203 DEGs showed that the TEP RNA could satisfactorily distinguish RCC and non-RCC individuals with only minor overlaps (**Figure 2C**). Besides, the DEGs in our dataset were also compared with the DEGs reported in NSCLC described by Best et al. (18), GBM described by Sol et al. (22), and PTC described by Shen et al. (20). The results showed that different tumor types only have a minor overlap of platelet DEGs, suggesting that the effects of the tumor on TEP RNA probably depend on the specific type of tumor (**Figure 2D**).

**Figure 3:**
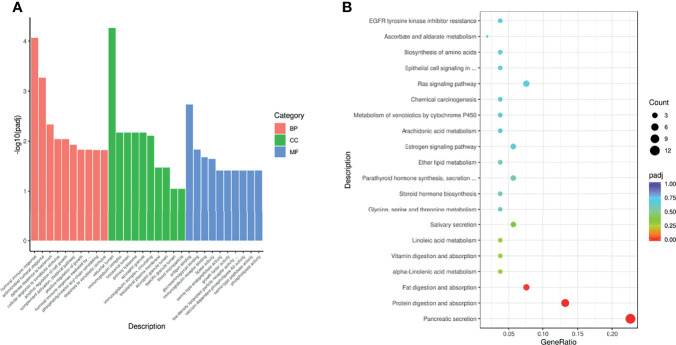
GO **(A)** and KEGG **(B)** enrichment analysis of DEGs between the RCC and control groups.

The GO analysis revealed that DEGs were enriched in three main categories, namely, “biological process”, “cellular component”, and “molecular function” (**Figure 3A** and **Supplementary Table 3**). The DEGs in the biological process were enriched in several pathways including humoral immune response, antimicrobial humoral response, and defense response to bacterium. Besides, the cellular component category contains several GO terms such as vacuolar lumen, immunoglobulin complex, and lysosomal lumen, while the molecular function category contains the terms antigen binding, glycosaminoglycan binding, and immunoglobulin receptor binding. The KEGG pathway enrichment analysis showed that the DEGs were mainly enriched in pancreatic secretion, protein digestion and absorption, and fat digestion and absorption (**Figure 3B** and **Supplementary Table 4**).

### Development and Validation of the Pan-Cancer TEP Model

**Figure 4:**
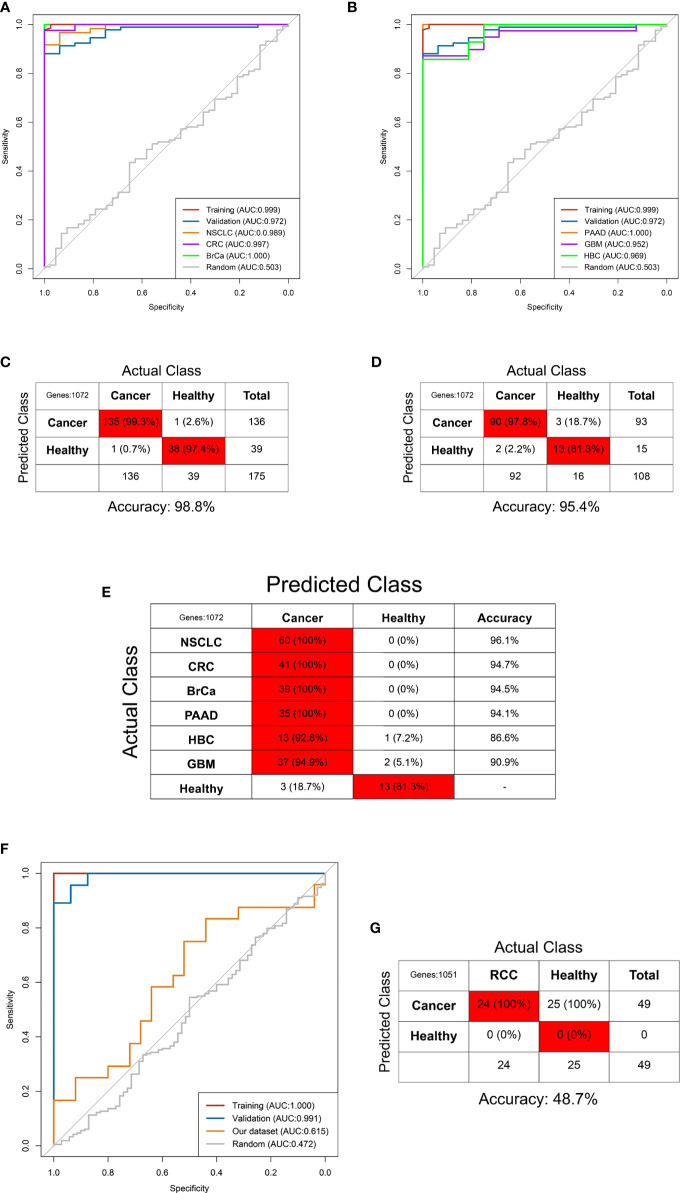
Predictive accuracy of the pan-cancer TEP model shown by the ROC curves in the internal **(A, B)** and external validation **(F)** and the confounding matrix in the training **(C)**, validation **(D)**, and internal different tumor types **(E)** and external RCC patients **(G)**.

The GSE68086 database contains 228 pan-cancer samples and 55 HD samples, including 139 pan-cancer samples and 39 HD in the training set and the remaining in the validation set. The SVM algorithm containing 1,072 DEG features was trained using the sample from the training set by a 10-fold internal validation to develop the pan-cancer TEP model. The best gamma and cost parameters were identified as 0.001 and 10, and then, the SVM parameter was locked. Subsequently, the pan-cancer TEP model was validated in both the training and validation sets. The AUC of the model in the training and validation sets was 0.999 and 0.972, respectively (**Figures 4A, B**). Besides, the sensitivity, specificity, and accuracy in the training set were 99.3%, 97.4%, and 98.8%, respectively, while in the validation set, these were 97.8%, 81.3%, and 95.4%, respectively (**Figures 4C, D**).

Next, the pan-cancer dataset was separated into NSCLC, colorectal cancer (CRC), pancreatic cancer (PAAD), breast cancer (BrCa), hepatobiliary cancer (HBC), GBM, and HD subgroups to internally validate the predictive value of the pan-cancer TEP model in detecting cancer in different tumor subtypes. These subgroups were validated using the pan-cancer TEP model. The results revealed that the pan-cancer TEP model could also detect the specific tumor contained in the model development. The AUC of the model for different tumor types ranged from 0.952 to 1.000 (**Figures 4A, B**), and the accuracy ranged from 86.6% to 96.1% (**Figure 4E**). The model was also used to detect RCC using our database to externally validate the performance of the pan-cancer TEP model in RCC. The accuracy and AUC of the pan-cancer TEP model for RCC were only 48.7% and 0.615, respectively, suggesting that the pan-cancer TEP model had a scarce diagnostic value in detecting RCC (**Figures 4F, G**).

### Development and Optimization of the RCC-Based TEP Model

**Figure 5:**
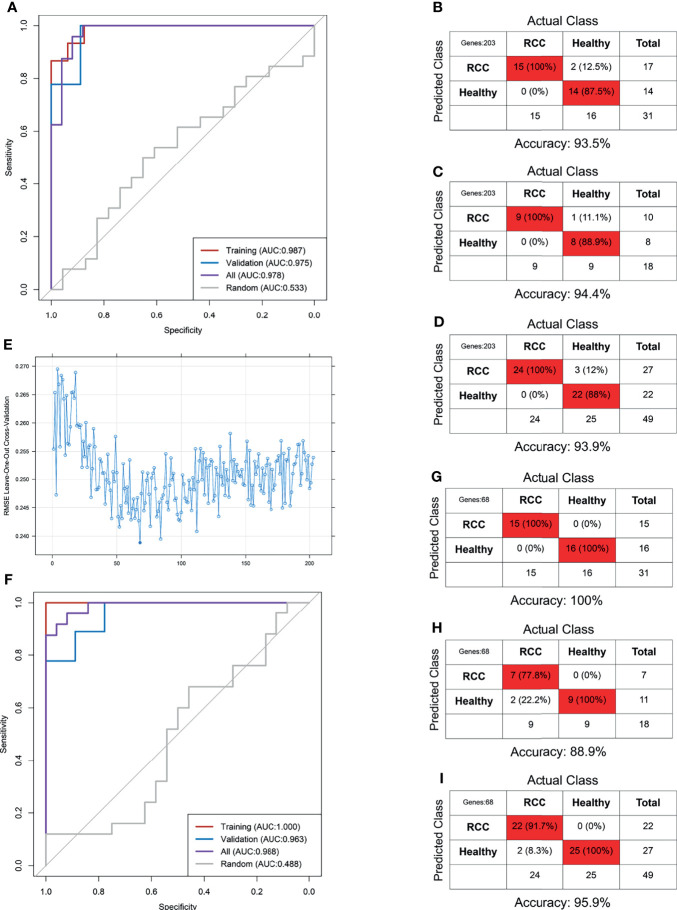
The RCC-based TEP model for the detection of RCC. The ROC curve **(A)** and the confounding matrix in the training set **(B)**, validation set **(C)**, and overall cohort **(D)** of the primary model. The ROC curve **(F)** and the confounding matrix in the training set **(G)**, validation set **(H)**, and overall cohort **(I)** of the optimized model after REF selection **(E)**.

Our dataset including 24 RCC and 25 non-RCC was randomly divided into training and validation sets at a 6:4 ratio. The RCC-based TEP model was preliminarily developed using the 203 DEGs in the training set. The best gamma and cost identified were 0.00001 and 100 after the 10-fold internal validation. The primary RCC-based TEP model showed an excellent performance in the training set, validation set, and overall cohort, with an AUC of 0.987, 0.975, and 0.978, respectively (**Figure 5A**). Besides, the sensitivity, specificity, and accuracy in the training set (100%, 87.5%, and 93.5%), validation set (100%, 88.9%, and 94.4%), and overall cohort (100%, 88%, and 93.9%) were also favorable (**Figures 5B–D**). Next, the RFE method was used to select the optimal biomarkers to achieve a high prediction and few DEGs. After RFE selection, 68 DEGs were chosen as the optimal biomarkers in the RCC-based TEP model (**Figure E** and **Supplementary Table 5**). Then, the optimized RCC-based TEP model was developed in the training set and validated in the training set, validation set, and overall cohort (gamma and cost were 0.001 and 10). Finally, the AUC of the optimized model was 1.000, 0.963, and 0.988, respectively (**Figure 5F**). The sensitivity, specificity, and accuracy in the training set (100%, 100%, and 100%), validation set (77.8%, 100%, and 88.9%), and all cohorts (91.7%, 100%, and 95.9%) were also satisfying (**Figures 5G–I**). In the subgroup analysis, the RCC-based TEP model also showed a high accuracy in detecting both local (accuracy: 95.2%, AUC:0.991) and metastatic RCC (accuracy: 100%, AUC:1.000) (**Supplementary Figure 4**). The optimized RCC-based TEP model was comparable to the first model but with fewer biomarkers, and an external validation was easier to be performed.

## Discussion

Platelets are non-nucleated blood cells in the human circulation and cannot synthesize RNA on their own. The RNA repository of platelets including mRNA, miRNA, and lncRNA was mainly derived from megakaryocytes, and some of them are endocytosed from circulation. Tumor cells can directly bind to the platelets through selectin-P and glycoprotein-IIa/IIIb to directly “educate” platelets to participate in tumor progression and metastasis, or indirectly educate them by secreting extracellular molecular substances such as thrombin, tissue factor, matrix metalloproteinase, and ADP (29–31). This “education” process also causes specific changes in the RNA profile of the platelets. Preclinical studies showed that tumor cells may affect the RNA profile of the platelets through the following mechanisms (32): 1) secreting tumor-derived cytokines that affect the maturation of megakaryocytes, 2) inducing protein translation in the platelets and accelerating the degradation of RNA, 3) stimulating specific splicing events of the pre-RNA in the platelets, and 4) sequestrating the tumor-derived RNA. The above mechanisms suggest the presence of a highly dynamic RNA profile in the platelets, which makes them feasible for liquid biopsy in the detection of cancer.

Calverley et al. (33) were the first to discover that the mRNA profile of the platelets in patients with metastatic lung cancer is significantly altered compared with that in the HD, with 3 upregulated genes and 197 downregulated genes. Besides, Nilsson et al. (34) revealed a distinct RNA signature in platelets from glioma patients compared with that from healthy individuals. They also discovered that platelets isolated from glioma and prostate cancer patients contain the cancer-associated RNA biomarkers EGFRvIII and PCA3, respectively. Subsequently, they enrolled 77 patients with NSCLC and found that platelets can be used for the non-invasive detection of EML4-ALK rearrangements in these patients predicting the outcome of the therapy (35). Another study demonstrated that the RNA of the platelets can also predict the response to the therapy with abiraterone in patients with castration-resistant prostate cancer (36). These results suggest that platelet RNA partially reflects the molecular characteristics of a primary tumor and predicts the therapeutic response to a specific drug.

The development of second-generation high-throughput sequencing technology allowed Best et al. (17, 18) to bring the concept of TEPs to the forefront of “liquid biopsy” of the tumor through a series of exploratory studies. In 2015, they prospectively enrolled 228 pan-cancer and 55 HD with RNA-seq on platelet samples, discovering that TEPs allow not only the detection of cancer when present (accuracy 96%) but also the evaluation of the type of cancer (accuracy 71%) across six different types. They also expanded the study cohorts to further explore the diagnostic value of TEPs for NSCLC, including 779 NSCLC patients and 339 controls, and they optimized the algorithm (18). Their results using the particle-swarm optimization-enhanced algorithms demonstrated that TEPs enable the detection of early (accuracy 81%) and late (accuracy 88%) NSCLC, regardless of the age of the individuals, smoking habits, whole-blood storage time, and various inflammatory conditions (18). After the protocol was reported by Best et al. (23), several studies explored the diagnostic value of TEPs in patients with sarcoma, PTC, ovarian cancer, and GBM, with an excellent predictive accuracy (19–22).

Despite that, to our knowledge, evidence that TEPs could serve as a diagnostic tool in patients with RCC is still lacking. The aim of this study was to investigate the diagnostic value of TEPs in patients with RCC, and several noteworthy findings were found and described. Firstly, the TEP transcriptome was significantly altered in patients with RCC compared with that in controls. The alteration in TEPs was different compared with other cancers and the majority of the alteration was exclusive, which was consistent with the findings reported by Heinhuis et al. (19). Secondly, the pan-cancer TEP model had an excellent performance in detecting patients with pan-cancer and specific cancer contained in the model development, but it could not be used to distinguish those with RCC. To our knowledge, the pan-cancer TEP model contains six types of tumors but without RCC. The low diagnostic accuracy of the pan-cancer TEP model for RCC patients also explains that different tumors had heterogeneous effects on the platelet RNA, and the pan-cancer TEP model is not universal for all cancers. The TEP model should be cancer-specific. Eventually, the TEP model for RCC patients was developed and optimized with satisfactory accuracy. It is worth noting that the model only contains 68 gene signatures, but the accuracy was comparable to other TEP models reported in the above cancers. Our hypothesis is that TEPs could be used as a promising tool for liquid biopsy in RCC populations, although an external validation is still needed.

This study has some limitations. Firstly, the sample number is relatively low especially when divided into training and validation sets due to the prospective nature of the study. Thus, more samples are needed to develop a more robust prediction algorithm. Secondly, the TEP model was not externally validated in another RCC cohort, potentially leading to an overestimation of the predictive accuracy. Thirdly, although the TEP model demonstrated high predictive accuracy in our analytic cohorts, more than half of the enrolled patients had cancer at an advanced stage (T3–T4). Thus, it is necessary to include more patients with low-stage RCC to verify our findings in a prospective study. Finally, the potential role of TEPs as a universal biomarker still needs to be verified in additional different types of tumors, and the function of altered RNAs in platelets still needs to be investigated.

## Conclusion

Our dataset provided a preliminary reference and resource for the TEP RNA profile in patients with RCC. Our results demonstrated that the pan-cancer TEP model barely detected RCC. Thus, the RCC-based TEP model was developed using our dataset with high accuracy in cancer detection.

## Data Availability Statement

The original contributions presented in the study are publicly available. These data can be found here: NCBI, PRJNA799249, https://www.ncbi.nlm.nih.gov/bioproject/PRJNA799249.

## Ethics Statement

The studies involving human participants were reviewed and approved by Peking University Third Hospital Medical Science Research Ethics Committee (IRB:00006761). The patients/participants provided their written informed consent to participate in this study.

## Author Contributions

Study concept and design: LM and BZ. Analysis and interpretation of data: RX and CL. Drafting of the manuscript: RX and CL. Critical revision of the manuscript: LM. All authors read and approved the final manuscript.

## Funding

This work was supported by grants from the Natural Science Foundation of China (82070778, 81972381, 82173385).

## Conflict of Interest

The authors declare that the research was conducted in the absence of any commercial or financial relationships that could be construed as a potential conflict of interest.

## Publisher’s Note

All claims expressed in this article are solely those of the authors and do not necessarily represent those of their affiliated organizations, or those of the publisher, the editors and the reviewers. Any product that may be evaluated in this article, or claim that may be made by its manufacturer, is not guaranteed or endorsed by the publisher.

## Supplementary Material

Supplementary Figure 1 The purity of platelets of 6 different samples **(A–F)** in morphological analysis after two-steps configuration (Wright-Giemsa stain, 400×).

Supplementary Figure 2 Gene expression analysis of platelet samples. **(A)** Gene expression box plot; **(B)** Principal component analysis plot; **(C)** Sample correlation heatmap matrix.

Supplementary Figure 3 Analysis of differentially expressed genes (DEGs) in the platelet of local **(A)** and metastatic **(B)** RCC compared to control group.

Supplementary Figure 4 The optimized RCC-based TEP model for the detection of RCC. ROC curve **(A)** and confound matrix in local **(B)** and metastatic **(C)** RCC.
