# Supplementary figures and images for: Tumor-Educated Platelets as a Promising Biomarker for Blood-Based Detection of Renal Cell Carcinoma
Source: Front Oncol. 2022 Mar 7;12:844520. doi: 10.3389/fonc.2022.844520 (PMC8936192; doi:10.3389/fonc.2022.844520)

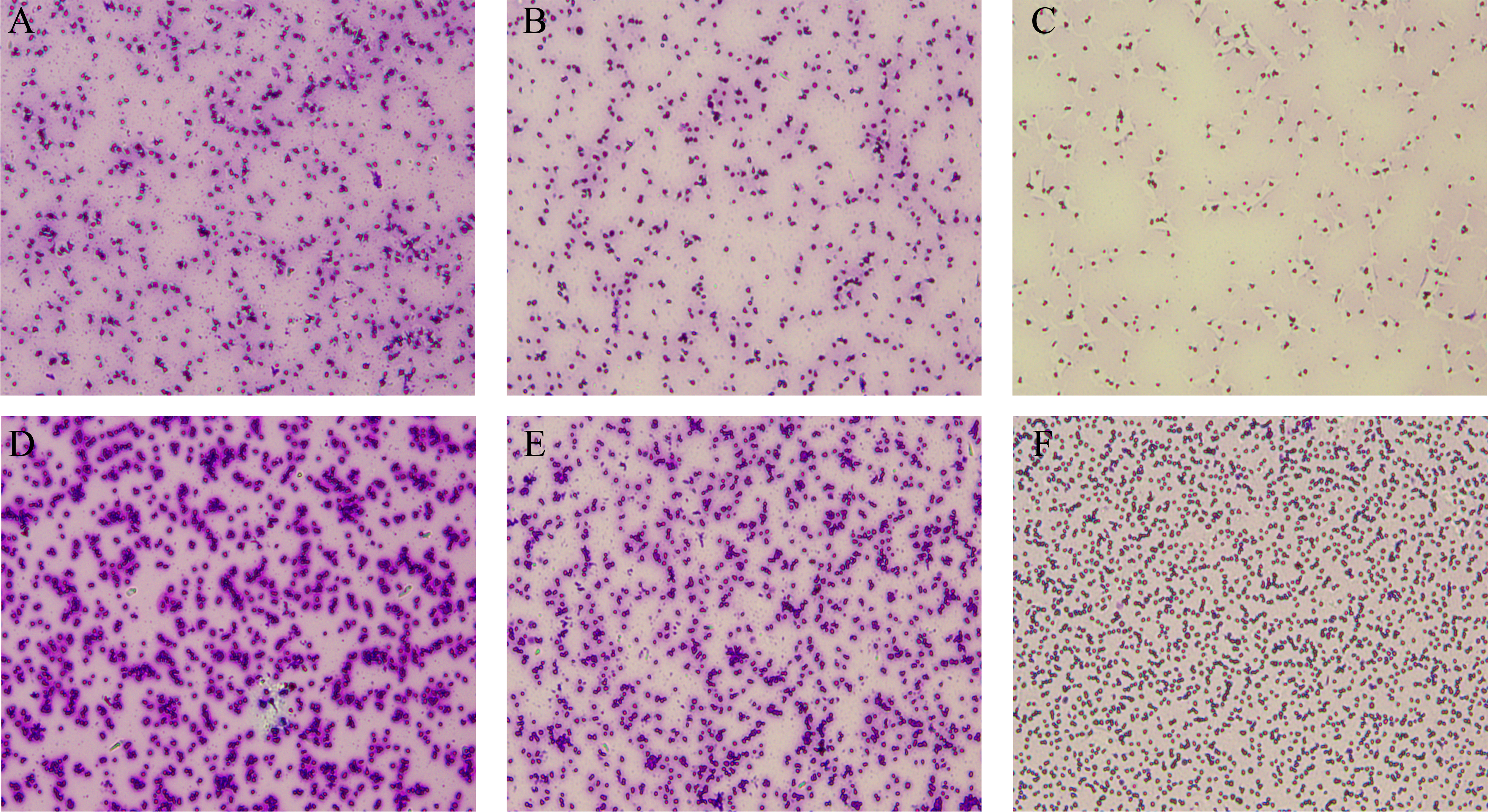

Supplement: Supplementary Figure 1 — The purity of platelets of 6 different samples (A–F) in morphological analysis after two-steps configuration (Wright-Giemsa stain, 400×). [file Image_1.tif]

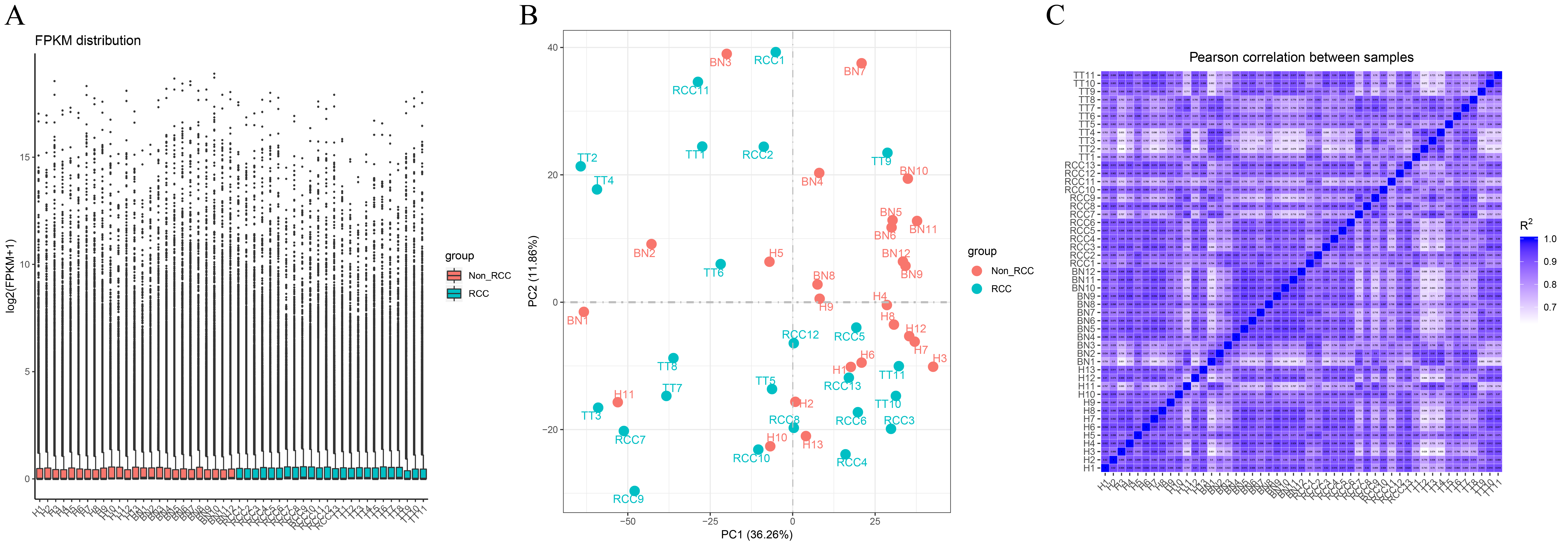

Supplement: Supplementary Figure 2 — Gene expression analysis of platelet samples. (A) Gene expression box plot; (B) Principal component analysis plot; (C) Sample correlation heatmap matrix. [file Image_2.tif]

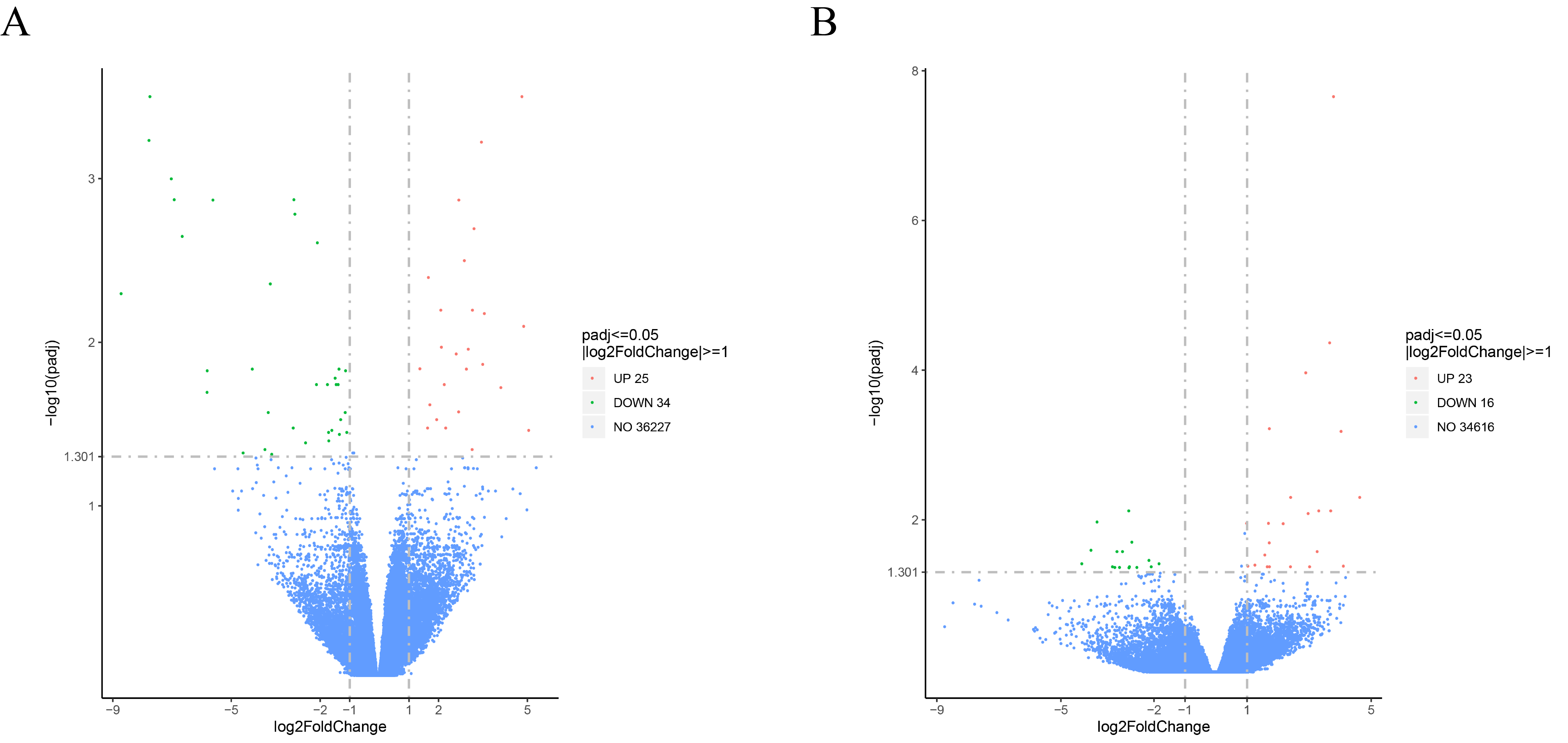

Supplement: Supplementary Figure 3 — Analysis of differentially expressed genes (DEGs) in the platelet of local (A) and metastatic (B) RCC compared to control group. [file Image_3.tif]

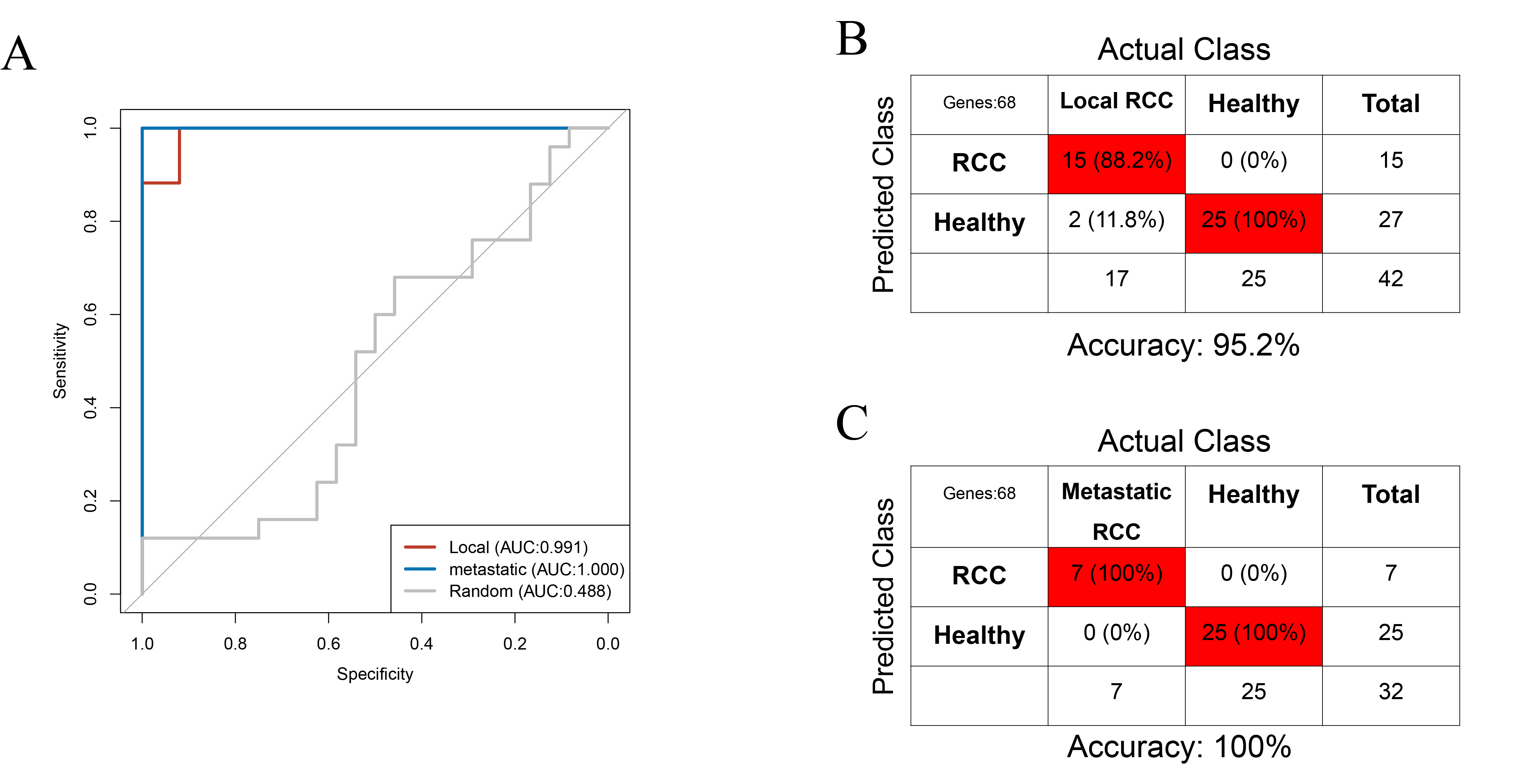

Supplement: Supplementary Figure 4 — The optimized RCC-based TEP model for the detection of RCC. ROC curve (A) and confound matrix in local (B) and metastatic (C) RCC. [file Image_4.tif]
