# Supplementary material for: Tumor-Educated Platelets as a Promising Biomarker for Blood-Based Detection of Renal Cell Carcinoma
Source: Front Oncol. 2022 Mar 7;12:844520. doi: 10.3389/fonc.2022.844520 (PMC8936192; doi:10.3389/fonc.2022.844520)
Supplement: Supplementary file 9 [file Table_5.docx]

Table S5. The 68 gene biomarkers for detection of RCC after REF selection

| Gene name/ID | | | | |
| --- | --- | --- | --- | --- |
| CPA1 | CELA2A | AGR2 | AC009927.1 | AL157700.1 |
| PNLIP | RARRES2P2 | CELA3B | NCAM1-AS1 | DPY19L1P2 |
| AMY2A | DDX11L5 | CTRB1 | DCN | AC024451.4 |
| CPB1 | AC135893.1 | SIAH2-AS1 | CELA2B | novel.963 |
| CLPS | PLA2G1B | AC087284.1 | PLA2G2A | novel.625 |
| AC026979.2 | RFPL3 | AL513314.2 | SYCN | novel.463 |
| GP2 | ST13P18 | LRFN2 | GATA6-AS1 | novel.263 |
| GABARAP | TMEM70 | LINC02849 | AC005307.1 | novel.240 |
| NPAP1P2 | DDX50P1 | TRDJ2 | AC099329.1 | novel.2100 |
| AC106795.3 | HMGN1P17 | AC023483.1 | AC105942.1 | novel.1832 |
| AL109955.1 | CEACAM5 | REG1A | AL133230.2 | novel.1656 |
| P2RY4 | AC139491.3 | ANKRD63 | FREM3 | novel.1259 |
| DHCR24 | ANKRD18DP | BDNF | AL354809.1 |  |
| S100A12 | PRRX1 | UGT2B28 | MEIS2 |  |
